# Human Biomonitoring in the Oil Shale Industry Area in Estonia—Overview of Earlier Programmes and Future Perspectives

**DOI:** 10.3389/fpubh.2020.582114

**Published:** 2020-11-12

**Authors:** Hans Orru, Anu Viitak, Koit Herodes, Triin Veber, Märten Lukk

**Affiliations:** ^1^Department of Public Health and Clinical Medicine, Umeå University, Umeå, Sweden; ^2^Institute of Family Medicine and Public Health, University of Tartu, Tartu, Estonia; ^3^Department of Chemistry, Tallinn University of Technology, Tallinn, Estonia; ^4^Institute of Chemistry, University of Tartu, Tartu, Estonia; ^5^Estonian Health Board, Tallinn, Estonia

**Keywords:** industrially affected lands, heavy metal, air pollution, PAH, HBM

## Abstract

Ida-Viru County, in Eastern Estonia, features industrially contaminated sites–where oil shale has been mined and used for electricity generation, and shale oil extraction. Higher prevalence of respiratory and cardiovascular disease has been found in the region due to high quantities of air pollution. Within the framework of “Studies of the health impact of the oil shale sector—SOHOS,” this analysis aimed to map earlier human biomonitoring (HBM) studies and identify the suitable biomarkers for upcoming HBM in Estonia. Altogether, three studies have been conducted among residents: first, among adults in the 1980's; second, among children in the 1990's; and third, among employees, with a focus on workers and miners in the oil shale chemistry industry in the late 1990's and 2000's. In some of those studies, increased levels of biomarkers in blood and urine (heavy metals, 1-OHP) have appeared; nevertheless, in last 20 years, there has been no population-wide HBM in Estonia. According to air pollution monitoring and emission analysis, the pollutants of concern are benzene, PM_10_, PM_2.5_, and PAHs. In general, there is a decreasing trend in air pollutant levels, with the exception of a slight increase in 2018. One of the aims of HBM is to be analyzed if this trend can be identified in HBM, using similar biomarkers as applied earlier. The future perspective HBM could be divided into two Tiers. Tier 1 should focus on exposure biomarkers as heavy metals, PAH, and BTEX metabolites and Tier 2, in later stage, on effect biomarkers as Ox LDL, TBARS, etc.

## Introduction

In Estonia, mining, and use of mineral resource oil shale for generating electricity generation and producing shale oil, is concentrated in Ida-Viru County– a large-scale industrial area in Eastern-Estonia. Oil shale is a fine-grained sedimentary rock containing organic matter, in the form of kerogen, which yields substantial amounts of oil and combustible gas. Oil shale industry activities have led to various environmental problems in the region, the most important of which is ambient air pollution. Frequently, those industry affected areas are called industrially contaminated sites (1), defined as “areas hosting or having hosted human activities which have produced or might produce environmental contamination of soil, surface or groundwater, air, food-chain, resulting or being able to result in human health impacts” (2).

Ambient air quality affects health, as toxic substances entering the respiratory tract can also reach other organs through the bloodstream, and cause various health problems and diseases (3). Health effects, related to the oil shale sector, have been studied in Estonia; studies suggest poorer health indicators, including increased respiratory and cardiovascular disease prevalence and a decrease in life expectancy (4). In addition, children living in the area have a higher risk of developing asthma (5, 6). Also a recent study has found the lung cancer age-standardized incidence rate being higher in Ida-Viru County compared to Estonia overall (7). One of the weaknesses in epidemiological studies has been poor exposure data, as discussed by Orru et al. (4, 6). We believe the implementation of human biomonitoring (HBM), would help to specify data on exposures in the population and advantage to refine the results.

Within the framework of “Studies of the health impact of the oil shale sector—SOHOS,” the current analysis aims to map the earlier HBM studies in the oil shale sector area, as well as elsewhere in Estonia, and identify the suitable biomarkers with an appropriate analysis methodology for the upcoming HBM program in Estonia.

## Human Biomonitoring

### The Human Biomonitoring Methodology

HBM is a methodology for assessing human exposure to natural and man-made compounds from living or working environments. Typically, specific substances or their degradation products, called metabolites, are measured in blood, urine, breast milk, and other body fluids or human tissues. The most commonly used and preferred biological matrix for HBM is blood, as it is in contact with organs and tissues where many chemicals are deposited (8, 9). However, there is a growing interest in non-invasive biomarkers, e.g., urine, that allow more routine sampling in human studies and reduce the number of blood sample refusers (9). Accordingly, previous studies of the health effects of the oil shale sector have used fractional exhaled nitric oxide (FeNO), which is a biomarker of airway inflammation (5, 6).

Compared to environmental monitoring, HBM has several advantages. For example, biological samples can characterize repeated exposure and the interaction of different exposures. HBM directly describes contaminants that have entered the body from all routes of exposure—inhalation, dermal absorption, and ingestion– and reflects individual differences due to different exposure levels, metabolism, and excretion rates. HBM data also refers to human physiological variations such as bioavailability, bioaccumulation, and persistence, which may increase the levels of some environmental chemicals (e.g., persistent organic pollutants) (9–11). Nevertheless, different matrixes (blood, urine, hair, breast milk, etc.) and different chemical physicochemical characteristics might characterize different exposures. If chemical substances, or their metabolites, are released rapidly from the human organism, repeated sampling at the individual level– to indicate a long-term exposure pattern– is needed (12).

The generally accepted classification of biomarkers divides them into two main categories: (1) exposure biomarkers and (2) effect biomarkers. Exposure biomarkers detect and measure chemical residues, or metabolites, in tissues or body fluids. It has been found that the advantage of utilizing exposure biomarkers is the so-called integrated measurement exposure, which is especially important in the case of substances with large differences in absorption depending on the time and location of exposure (9).

Effect biomarkers measure the processes that are considered to be “early events” associated with disease-related changes (13). An important group of effect biomarkers is genotoxicity biomarkers in workers or, to a lesser extent, in the population exposed to mutagens or genotoxic carcinogens. Many tests are used for the detection of DNA damage, such as micronucleus counting, Comet Assays (single cell gel electrophoresis), chromosomal aberrations, DNA adducts, etc (14).

However, effect biomarkers have been found to be effective only when people exposed to high levels of contaminants (e.g., working with mutagenic agents), and they are difficult to use to differentiate the effects of individuals. Toxicological studies have shown that individuals' responses to chemical exposure can often vary significantly (15). Ladeira and Viegas (14) consider that such differences between individuals may be genetically mediated or caused by some environmental stressor, disease process, or other epigenetic factor. Thus, effect biomarkers are currently recommended to be used as group indicators—they are sensitive, but not contaminant-specific and often difficult to interpret. Effect biomarkers have been used in earlier studies among oil shale sector workers (see chapter HBM Among Employees in the Oil Shale Sector for details).

### National HBM Programs and HBM4EU Network

Many countries have established national HBM programs. The main objectives of these programs are to develop and validate biomarkers based on certain exposures and to predict the risk of disease for both populations and/or, under certain conditions, individuals (16). HBM can also identify spatial and temporal trends in human exposure as well as provide information on risk assessment. On the basis of risk assessments, decision-makers can be informed about chemical risks and policy measures can be initiated in order to protect population with the special focus on susceptible groups such as children and pregnant women (9, 11, 17). Successful examples of the impact of HBM include the banning of lead in petrol, avoiding mercury-containing amalgam teeth fillings, restricting the use of phthalates in plastics, and several other initiatives (18).

Within the Horizon2020 European HBM project HBM4EU has been established. It is now a joint project of 30 countries (Estonia joined in 2020), the European Environment Agency, and the European Commission for the period of 2017–2021 (19). The aim of this initiative is to promote and harmonize HBM activities in Europe, with the special focus to develop European HBM indicators (20). The harmonization of biomarkers collection and analysis– as well as the selection of which biomarkers to use– should make studies more comparable and give valuable reference material. Besides this, several other tools, such as questionnaires, have been developed which can be used in further HBM studies, i.e., in an Estonian HBM program that includes oil shale area (19).

## Air Quality in Oil Shale Industry Area

**Figure 1:**
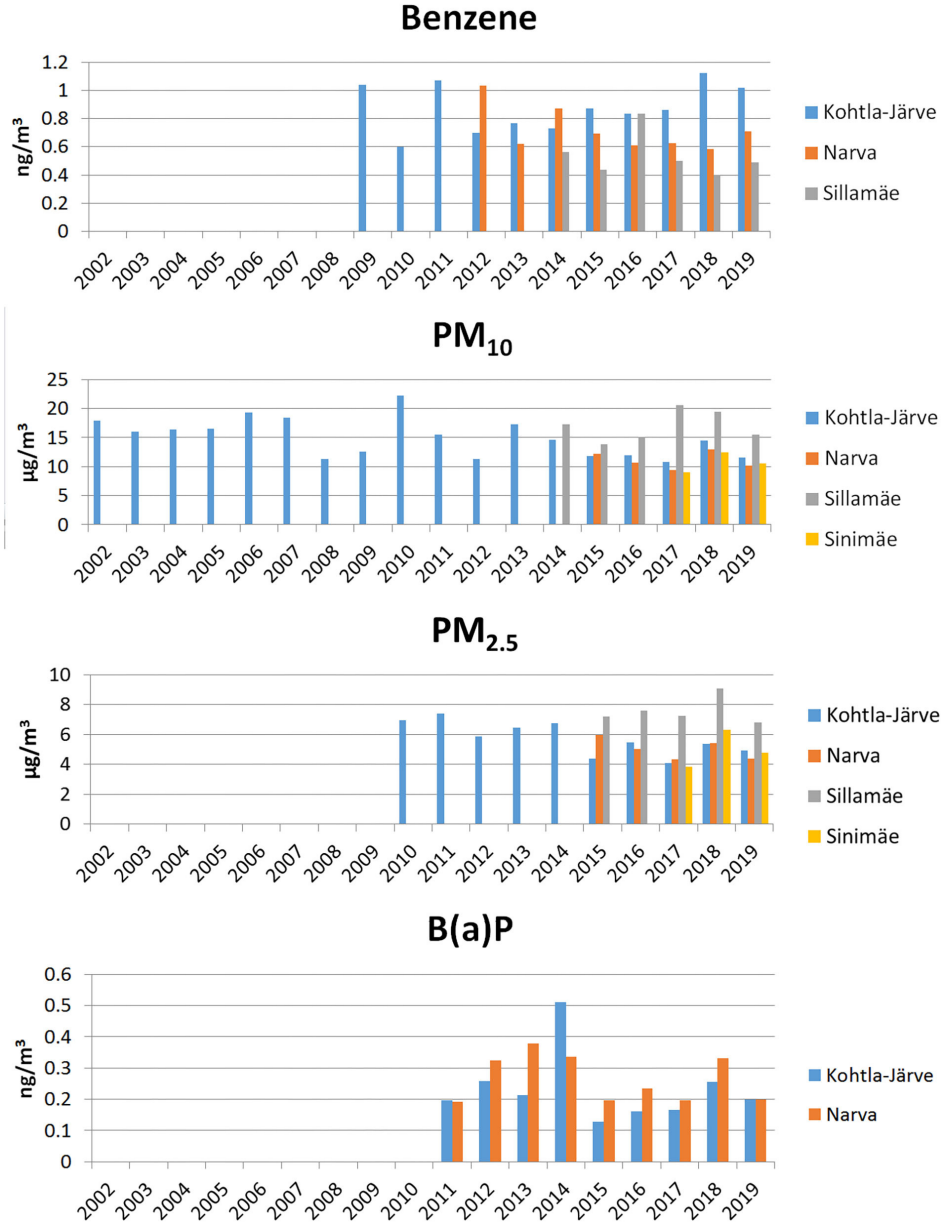
Annual average concentrations of benzene, particulate matter (PM_10_), fine particles (PM_2.5_) and benzo(a)pyrene (BaP) in monitoring stations in Ida-Viru County.

Previous studies have shown that the most important oil shale sector pollutants– that cause toxic and allergic reactions– are benzene, formaldehyde, phenol, particulate matter (PM_10_), fine particles (PM_2.5_), and polycyclic aromatic hydrocarbons (PAHs), including benzo(a)pyrene (B(a)P) (4–6). In Ida-Viru County, the air quality has been monitored in the cities of Kohtla-Järve, Narva, and Sillamäe, and in the Sinimäe region (Figure 1). In general, the air quality currently is said to be “good,” with few exceedances of pollutants' limit values in recent years (21, 22). However, several studies have indicated that the health effects of PM_10_ and PM_2.5_ also occur at concentrations below the set limit values (3), so health risks associated with air pollution still remain (4–6).

If we look at the long-term trends of pollutant concentrations, a slight decrease can be seen in the case of benzene in Narva, while in Kohtla-Järve the concentration has remained the same and in Sillamäe fluctuated from year to year (Figure 1). In the case of PM_10_ and PM_2.5_, the concentrations have decreased somewhat over time, but spiked in 2018 (Figure 1). Concentrations of B(a)P have been on a slight upward trend in 2011–2014, then decreased sharply in 2015, and then started to increase again (Figure 1). In the analysis of concentrations of heavy metals and PAHs, bound to PM_2.5_, we noted arsenic and cadmium remain at the same level in 2018–2019, whereas lead and nickel increased in 2018 in Narva and Kohtla-Järve, while both decreased again in 2019.

## Earlier HBM Studies in Estonia

In our analysis, we conducted three larger HBM studies among residents and three among employees. The residents' studies were country-wide (incl. oil-shale area) with focus on adults and children; occupational studies focused on oil-shale sector employees.

### HBM Among Residents

The largest HBM program took place in 1982–1985, where the content of heavy metals in the blood and hair of adults was studied in nine different regions in Estonia (Table 1). The study included residents aged 20–60 who had lived in the area for at least 5 years and were born, or lived, in Estonia for at least 20 years (23). In addition, later the data of residents from Maardu and Kostivere was analyzed (24).

**Table 1:** Average content of heavy metals in blood (μg/100 ml) and hair (μg/g) [based on (23–26)].

	**Lead**			**Cadmium**			**Mercury**			**Copper**	**Zinc**
	**Blood**		**Hair**	**Blood**		**Hair**	**Blood**		**Hair**	**Blood serum**	**Blood serum**
	**Adults**	**Children**	**Adults**	**Adults**	**Children**	**Adults**	**Adults**	**Children**	**Adults**	**Children**	**Children**
*Cities in oil shale region*											
Kohtla-Järve	17.12^3^	–^2^	11.07	0.96^3^	–^1^	2.37	0.53^1^		0.77		
Sillamäe 1990/94^*^		9.0/5.5			0.54/0.18					80.2/59.9	156.2/130.4
Narva		–^2^			–^2^						
*Country-side affected by cement production*											
Lahemaa	22.23^3^		12.83	0.47^2^		1.08	0.83^3^		0.65		
*Capital city*											
Tallinn	8.25^3^	–^1^	8.67	0.48^2^	–^0^	1.30	0.37^1^		0.62		
*Industrial complex near Tallinn*											
Maardu	16.0^4^		13.1	0.80^2^							
Kostivere	13.3^3^	–^1^	10.4	0.68^1^							
*Southern Estonia*											
Tartu 1990/94^*^		4.5/ 2.5^1^			0.70/ 0.17^1^					65.0/ 90.9	163.3/157.8
Võru	7.41^3^	–^1^	8.58	0.34^2^	–^0^	0.82	0.36^1^		0.84		
*Western Estonia and islands*											
Saaremaa	13.87^3^		10.80	0.57^2^		1.33	0.65^2^		1.90		
Haapsalu	12.52^3^	–^0^	11.13	0.32^1^	–^0^	0.65	0.82^3^		3.09		
Hiiumaa	9.15^2^		7.24	0.23^2^		1.27	0.49^2^		2.30		
Matsalu	8.22^1^		2.20	0.24^1^		0.30	0.79^3^		2.52		
Viidumäe	9.46^2^		5.07	0.21^1^		0.54	0.46^2^		1.14		

4*>2 times of “limit value,” ^3^1–2 times of “limit value,” ^2^½−1 times of “limit value,” ^1^½–¼ times of “limit value,” ^0^<¼ times of “limit value,” __0,1,2_ no exact value provided, only compared with “limit value”*.

* *Original data based on archive materials*.

The other two surveys were conducted among children in 1989–1990 and 1991–1994. In the first study (25), an increase in the concentration of some microelements in blood of oil shale region children was found according to the contamination of the external environment. The blood lead levels found in the children from Kohtla-Järve, Narva, and Sillamäe were on average three times higher than in Tartu (reference area). The cadmium concentration was 1.6 times higher in Kohtla-Järve compared to Tartu. The lead and cadmium levels in the blood of children from Kohtla-Järve were significantly higher compared to children from other two studied cities in Ida-Viru County. Compared to Tartu, the blood heavy metal concentrations were higher in all three Ida-Viru County cities. The second study (26) showed higher levels of lead and cadmium in children's hair in North-Eastern Estonia and higher levels of mercury in Western Estonia (in the hair of island and coastal residents). The higher levels of mercury are most likely related to the consumption of fish caught in the Baltic Sea. In that study, the values were also compared to limit values at that time. The lead values in blood were exceeded allowable limit of that time (27) in almost all regions, whereas cadmium levels were exceeded only in Kohtla-Järve and mercury levels in Western-Estonia and Lahemaa (affected by cement factory pollution).

### HBM Among Employees in the Oil Shale Sector

The occupational studies have focused on miners and coke oven, and benzene plant workers. Due to weak ventilation systems and diesel-powered machinery, miners had a significantly higher exposure to benzene than oil shale sector's surface workers. In 2000, the concentration of benzene in the oil shale mine was 190 μg/m^3^, while in the urban air of Kohtla-Järve the concentration of benzene was 29 μg/m^3^ (28). Furthermore, mine workers are exposed to elevated levels of PM_2.5_ and PAHs, which has resulted in 7.5 times higher levels of 1-nitropyrene (1-OHP) compared to ground workers (29, 30).

Of these studies, the first HBM looked at the exposure of coke oven workers to PAHs (31, 32). The workers were highly exposed, as the average amount of B(a)P in the air inhaled by workers was 5.7 μg/m^3^ (32). The study also measured the urinary concentrations of 1-OHP and white blood cell DNA adducts. Though 1-OHP levels correlated with the number of DNA adducts, coke oven workers did not differ significantly compared to the controls from nearby Iisaku village (33). The subsequent analysis found that genotype might have played an important role regarding both biomarkers (1-OHP and DNA adducts), as evidenced by significant differences in number of adducts by genotypes among coke oven workers (34).

The second study compared benzene plant and coke oven workers. Higher benzene concentrations were observed at the benzene plant, which was confirmed with personal monitoring of benzene in the exhaled air (35). The same study also found higher levels of benzene in the workers' blood and elevated levels of the metabolite t,t-muconic acid (MA) in the workers' urine. The later analysis of effect biomarkers did not indicate an increase in the number of micronuclei in buccal cells (36) or increased cancer-specific ras (p21) proteins in plasma (37). A subsequent study of chromosomal aberrations did not show significant differences between the serum albumin levels of the controls from nearby countryside residents and the people working at the Kohtla-Järve chemical plant; nevertheless, the levels of S-phenylcysteine adducts were considered relatively high in both groups (38).

The third study compared 50 underground mine workers exposed to diesel exhaust with 50 above-ground oil shale sector workers. It was found that miners had a higher degree of DNA damage (Comet assay data) compared to the control group (39). They had also several changes in the levels of biomarkers such as S-phenylmercapturic acid (S-PMA) and MA (28) and increased 5-aminolevulinic acid (ALA) activity (40). Muzyka et al. (40, 41) have claimed that exposure to diesel exhaust has caused changes in heme synthesis, resulting in the accumulation of ALA and protoporphyrin in miners' lymphocytes.

## Selection of Biomarkers for Estonian HBM

Based on the analysis of pollutants, emission and monitoring, the pollutants characterizing the environmental pollution of the oil shale sector would be PM_10_, and PM_2.5_ (as well as heavy metals bound to particles), benzene and PAHs such as B(a)P.

Elvidge et al. (42) have reviewed >20 biomarkers used as PM_2.5_ biomarkers. The most common of these are the markers of inflammation: C-reactive protein (CRP), interleukin 6 (IL-6), and fibrinogen. However, more than half of the studies using these markers have shown no effect. Less commonly used, but more robust biomarkers have been oxidized low-density lipoprotein (ox LDL), lipoprotein receptor-1, TBARS, which characterize lipid hyperperoxidation, and malondialdehyde (MDA), a marker of oxidative stress. However, the limitation is that these biomarkers are also elevated with cardiovascular diseases, so they are not specific for the contamination. Even more robust biomarkers are heavy metals bound to particles (43). The update of the temporal and spatial trends, should be encompassed. This should certainly include heavy metals from the earlier HBM in Estonia (lead, cadmium, mercury, copper, zinc), as well as arsenic and chromium.

In the case of benzene, we recommend to determine it as the BTEX (benzene, toluene, ethylbenzene, and xylene) metabolite complex, which has been used as a biomarker found in urine in petroleum distribution facilities (44). To assess exposure to PAH, it is valuable to use 1-OHP, which is the most commonly used PAHs biomarker in both work and living environments (45). It has been earlier used among oil shale sector workers (32).

## Chemical Analysis of Biomarkers

If the earlier studies mainly used different (AAS) techniques for heavy metal detection in blood and plasma, recently the Inductively Coupled Plasma Mass Spectrometry (ICP-MS) and Inductively Coupled Plasma—Optical Emission Spectrometry (ICP-OES) have been preferred due to much lower detection limits (46). ICP-MS and ICP-OES have been applied in several studies of populations living near industrially contaminated sites (47–49).

For 1-OHP determination HPLC with fluorescence detection (50), gas chromatography with mass spectrometry (GC/MS) (51), and more recently liquid chromatography tandem mass spectrometry (LC-MS/MS) (52) have been applied using isotopically labeled standard for 1-OHP detection (53). Another important metabolite would be 1-hydroxypyrene glucuronide (1-OHP-G), which allows better determination of low exposure to PAH (54). Due to low 1-OHP-G levels, more accurate ultra-high performance liquid chromatography–tandem mass spectrometry (UHPLC-MS/MS) should be applied (55).

For the determination of BTEX, several new studies have used headspace solid-phase microextraction (HS-SPME) coupled with gas chromatography-mass spectrometry (GC-MS) (56, 57). It is a sensitive methodology that can be used to determine low levels biomarkers. This is especially crucial among those less exposed, such as children (58).

## Discussion and Conclusions

HBM is valuable tool in indicating exposures as well as early effects in a large number of studies (9, 12). The information on human exposure can then be linked to data on sources and epidemiological surveys in order to inform research on exposure-response relationships in humans (19).

However, there are also several limitations in the interpretation of HBM results. One of the main limitations is that in many cases it may be difficult to confirm from which source the measured effects have been induced. This can be refined by using HBM and environmental monitoring data in parallel, where we can ultimately identify the sources of the pollutants.

Other major limitations are the confounding factors that could induce similar effects, e.g., in case possible biomarkers of PM_2.5_ health effects, existing cardiovascular disease might have a similar effect (42). One of the possibilities to collect data on confounding factors is through questionnaires.

Often, HBM is very costly and resource intensive. Tan et al. (59) have suggested that an HBM program could be divided into different tiers. We propose that Tier 1 could focus on exposure biomarkers as heavy metals, PAH, and BTEX metabolites. Tier 2, in a later stage, could focus on effect biomarkers, e.g., particle exposure biomarkers. Collecting blood samples in Tier 1 for Tier 2 could be beneficial.

Applying the described approached and taking into account the limitations, would warrant the solid ground for HBM in Estonia. Though, besides the exposed population, e.g., people living near oil shale industry or industry workers, the data from reference areas is needed in order to get good basis for spatial comparison. We support including the areas that have been analyzed earlier: Tallinn as capital area, Southern-Estonia as reference area and Western-Estonian coastal areas with somewhat different dietary habits like higher consumption of Baltic-Sea fish.

In the current analysis we could identify several HBM studies in the oil shale industry area in Estonia; however, all of those have been made in 1980, 1990, and 2000's. During the last two decades there has been change in environmental quality, so the update of HBM data is essential. As Estonia is recently joined HBM4EU network, this information would also be important from that perspective.

## Data Availability Statement

Publicly available datasets were analyzed in this study. This data can be found here: Data is available in the reports referred in the article.

## Ethics Statement

Ethical approval was not provided for this study on human participants because the current study only reviews earlier studies. The original studies had been approved by local ethics committees. Written informed consent to participate in this study was provided by the participants' legal guardian/next of kin.

## Author Contributions

HO and ML contributed conception of the study. HO and TV compiled the database of earlier studies. HO and AV contributed interpretation of the study findings. AV and KH reviewed the methodology used for chemical analysis in human biomonitoring. All authors contributed to writing the manuscript.

## Conflict of Interest

The authors declare that the research was conducted in the absence of any commercial or financial relationships that could be construed as a potential conflict of interest.
